# Lecture with Stereoptican—Dental Caries; Its Character, Cause and Effects

**Published:** 1899-01

**Authors:** O. W. Barrett


### THE DENTAL REGISTER.

Vol. LIII.] JANUARY, 1899. [No. 1.

### Communications.

### Lecture with Stereopticon—Dental Caries; Its Character, Cause and Effects.

BY PROF. C. W. BARRETT, M.D. D.D.S.

Given before the Tri-State Dental Meeting, at Put-in-Bay, June, 1898.

Mr. President, Ladies, and Gentlemen:—I do not know of any more important subject to dentists than that which is on the program for this evening. There is no more intense pain which man can suffer than that of toothache, and any one who has ever thus suffered has a full comprehension of the fact.

There are a good many things to be taken into account in the consideration of this subject. I wish to say a few words of general application, before proceeding directly to the theme of the evening.

What are teeth, in the first place? I dare say there are very many present who have never seriously considered what objects they are made to subserve, and what is really the character of the diseases to which they are liable.

In the first place, we know that teeth are not made primarily for mastication, because the great body of the animal kingdom, made up chiefly of the lower orders of animals, do not use them for that purpose. They have but one set of teeth, and use them primarily as organs for prehension—for catching and retaining their food.

The teeth of the serpents, for instance, are all alike and curved backward. When the snake has commenced the process of deglutition of the frog, it is utterly impossible for it to release its hold or for the frog to escape, if the snake happens to seize one of the legs first, this must be digested away before the teeth can release their hold and the frog seized in a position that allows it to be completely swallowed.

Teeth are also weapons of offence and defence, and perhaps there is no more important service to which they are put than this. They are used by some animals as tools for the construction of their habitations and for getting their food; and finally, teeth are used as organs of mastication, but only in the higher animals. In all the classes of animals, except birds, we find teeth. There are fossil birds which had full sets of teeth. Animals also have teeth distributed in different parts of the body. In the lower orders they are not confined to the maxillary bones but are found on the tongue, in the palate, pharynx, and some in the stomach. It is only in the higher orders, in animals with a heterodont-diphyodont dentition, that they are confined to the jaws. The teeth are differentiated in the different orders of animals, from the simple anchylozed homodont, almost innumerable teeth, and some fishes, and of the amphibia, up to the compound and highly organized, definite dentition of the ungulates of man. They vary in development and in specialization, from the great tusks of the mastodon or the elephant down to the odontophyore of the oyster. They are very widely distributed, all the classes of animals possessing them in some form, if as I have said we except birds, and even they have the horny mandible to serve in their place. Many imagine that diseased teeth are confined to man. A greater mistake could not be made; all of the diphyodont animals suffer from nearly all forms of dental diseases.

It has been supposed in the past that diseased teeth are of modern origin and are due to man's sins, because he has forsaken the dictates of nature and leads an artificial life. Many believe that if man could lay aside his civilization and his factious environments, and could go back to aboriginal life and live according to the rules of savage nature, he would never have diseases of the teeth. Some of the worst cases of irregularity and degeneracy of teeth I have ever seen have been in the lower orders.

They are frequent in the domestic animals, especially, and some of them are almost as common as in the human race.

Good old Dr. John Allen, some of us who are not boys remember him and the papers he used annually to present, in which he always spoke against the millers, who he affirmed were responsible for the degeneration of the teeth, as they removed from the grain that which should go to the making of the teeth and bones.

We now know that is all a mistake. I have skulls and single teeth nearly 4,000 years old, and they show precisely the same degeneracy that we see to-day. I have the skull of a gorilla which died in a state of nature and never used a tooth-brush, and it had the worst case of alveolar and antral abscess that I ever saw. I suppose he suffered from the toothache, and was just as disagreeable in domestic life as some of his congeners are to-day under the same circumstances. He probably kicked the furniture about the house and scolded his wife and cuffed the children because his tooth ached.

No, caries is not a modern disease; it dates back to the earliest period of man; as far back as we can trace human history we find men suffering from dental diseases, and we must remember in those early days they were not solaced by the soothing tenderness, the delicate companion, the sympathizing tenderness of the modern dentist and his tranquilizing dental engine and nerve broach. It must not be forgotten that the human teeth are developed from bone, differentiated to some extent. I will show you some of these upon the screen. Some of the slides may seem elementary to you, but remember there are those present to whom these matters are new, rudimentary as they may seem to some of my professional brothers. The teeth are the results of the evolution not only of species but of tissues and organs.

I am well aware that there are a few mistaken souls who value the creeds and dogmas of men far above absolute truth, to whom the doctrine of evolution is as a red rag is to a bull, yet it stands for all their want of faith. I have never known of but one man who was competent to express an opinion—that is, who really comprehended what evolution is—that dared deny it; that man has private and unworthy prejudices which he would not lay aside. The true theory of evolution is not the question whether or not man has descended from a monkey. Whoever makes that conclusion simply demonstrates that he does not know what evolution is. I am a firm believer in evolution, but I do not believe that man was ever a monkey or an ape or a hog or a long-tailed rat. Under divine guidance he has developed from a lower state of existence, and is yet advancing and progressing toward planes of development of which we at present have little conception. The truly intelligent man who thinks and reasons must inevitably reach certain conclusions, and the true theory of evolution is one of them. I do not teach the doctrine of man's descent from apes. Apes were always apes. Man was always a man from his first inception in the small organic cell, and he has simply developed from that point.

Bone cannot be exposed in any way without making a compound lesion. Enamel is constantly exposed. It is developed from bone through the intermediate stages of cementum and dentine, and its power of resistance is proportionate to its development.

The diseases to which teeth are liable have been a source of much speculation ever since man existed. I suppose that Adam had toothache. I presume it was the malic acid of that apple which dissolved out the lime salts of his tooth, and Eve was, of course, responsible for it. In the olden time it was claimed that decay was due to a worm, to inflammation, etc. Many theories existed, until 1750 it was claimed to be a true inflammation of the dentine. Another theory concerning the etiology of tooth caries that has been believed from the earliest etiological times until to-day holds that it is a chemical solution of the elements of the tooth. Dr. Watt, of honored memory, even supposed it to be due to mineral acids. There are certain dentists who believe to-day that electrolysis causes the solution of the elements of the tooth. Some of my older friends remember the days when we used to battle over the question of the causes of caries, but we could never positively demonstrate anything.

Finally, in 1881, at the time of the International Medical Congress in London, a paper was produced which was the seed of fact.

It was not the truth itself, but it contained the elements of reality, and it stimulated mental activity until the problem was solved. I heard that paper and was interested at the time. It dwelt on the bacteriological pathology. The cause was discovered by one who had studied in this country and then went abroad and there became interested in this unsettled question, and he never rested until he solved the problem. It is a rare thing to be able to say to the world, you are wrong and I alone am right. No; it is a very common thing, but it is a great thing to be able to demonstrate and prove it.

Dr. Miller, an American dentist in Berlin, who has refused the highest honors because he would not resign his American citizenship, was the man who solved the problem. He made one of the greatest discoveries of the age. Perhaps I ought not to dwell upon this subject, but your president informs me that I have the whole night at my disposal and I cannot forbear from adverting to this the grandest era in the history of dentistry.

Miller put into the solution of this mystery of the ages all his life, all that makes existence attractive, for he sacrificed ease and comfort and health in the pursuit of his investigations. He triumphed, but at a fearful cost, at the expense of that which neither applause of his fellow-practitioners, the honors of those in power, nor even the wealth of the Indies could compensate him.

I have not prepared any written address for this occasion, because I knew that it would be necessary to give the main part in darkness. Because I have not the time to give to the writing of a formal paper, as a consequence this address will be less systematic and consecutive, and I may at times repeat myself; this you must condone, remembering the exigencies of the occasion.

If now the lights can be turned out I will commence the exhibition of the illustrative stereopticon slides. I will begin by throwing upon the screen the classification of matter which I wish to follow—here it is:

Matter, { Organic, { Animal, Vegetable, { Phanerogams, Cryptogams

Inorganic,

---

Cryptogams { Acrogens, Thallogens, { Algar, Fungi, { Bacilli, Cocci, Vibrioues. Lichens,

It will be seen by this table that the fungi which are the organisms that we wish to study are strictly vegetable in their nature. I have often heard the bacteriological theory called the "bug theory." These organisms are as completely vegetable as a cabbage and there is no more animal about them than there is to a tomato. Matter is divided into two great sub-divisions, organic and inorganic. The latter concerns only inanimate things, so will leave that out of the discussion. Organic may be divided into animal and vegetable. I do not know where we can draw the absolute line between these two sub-kingdoms.

I have asked many a student to give me a perfect definition of man, of course metaphysical speculation is barred. What is man? What distinguishing characteristics does he possess that is not found elsewhere in any animal and which differentiates him from all animals? The only absolute difference which we know of is simply that man has two hands and two feet and the difference between a hand and a foot is the apposing thumb of the former. We can not draw the line at intelligence, for some animals know more than some men. Man walks upright; so does the chicken. I know nothing which distinguishes man except his two hands. The nearest congenes are the apes and monkeys which have four hands. But the organisms which we desire to consider are not animals, we will omit the latter and study vegetables.

All plants are divided into two great classes; Cryptogams, flowerless plants propagating by spores, and Phanerogam, flowering plants propagating by seeds. The Cryptogam is again divided into Acrogens, the higher flowerless plants containing cellular tissue only, such as ferns, mosses, etc., and Thallophytes or Thallogens, lichens, algae and the fungi.

The importance of the fungi is little comprehended. Some are so large and others so exceedingly minute. Some edible, mushrooms for example, are so large that one will make a meal for eight persons.

To one branch of the fungi belong those which we are to consider, the bacteria, which are microscopical in size. They occur in three forms—round, rod and screw. The round, cocci; the rod, bacilli, which may occur as clostridium and others; the screw forms, vibrio, spirilli, etc. Do not fail to remember that these organisms are of vegetable origin. I now present to you a slide exhibiting the relative arrangement of tissues of the human teeth. Here we have enamel prisms, there dentine and there cementum. I show you here a section of bone, because I wish to demonstrate to you that the tooth itself is but a differentation of bone.

In bone we have exactly the same elements as in teeth, but we have the lacunae arranged into a concentric form around some nutrient center and that distinguishes bone.

In the next slide we have a section of teeth exhibiting the cementum. You see that it has the lacunae of bone but there has been lost the regular concentric arrangement. Of course we have the canaliculi, but we can not see them plainly. In this slide we have dentine which has been raised to a higher power. It has lost the lacunae which contain the living matter, but there still remain the analogues of the canaliculi in the dental tubuli. In this slide showing enamel, we find we have even lost the canaliculi and there remains only the almost totally inorganic portion of bone as represented in the enamel prisms. Here we have a representation of a cross section of dentine. It apparently consists of a great number of tubes radiating through the body of the dentine. These tubules are filled with the so-called dentinal fibrillae, which make up the living portion of the dentine. No inorganic material can live in the body, it is all organic, it must have a vital connection and be of organic origin. Here is another slide showing a cross section of dentine. Part of these tubules have changed color through diseased action. Here are dental tubules in perfectly healthy condition. In that dark spot we see a modified area, which is diseased and which is easily distinguished from those in which there is no disease. There is something the matter with this, what is it? Let me here break off to explain what is the modern doctrine of the etiology of dental caries.

These bacteria of which I gave you the origin, have a peculiar function. It is that of tearing down that which nature builds up. The difference between death and life is loss of function. Wherever in any organic living body function ceases, then comes in the office of bactéria to tear down and decompose and liberate the elements and place them in such a condition that they may again be used. This small vegetable decomposes the substance; takes out that which it wishes, to build up its own organism and dismisses the other elements to form new combinations. They will perhaps appear in the form of grass; some animal eats the grass, and it is again through another process of fermentation caused to change into another structure. Hence, all flesh is, or has been, grass. The putrefaction of animal and decomposition of vegetable matter is all accomplished through the function of some fungi. The consequence of the growth of fungi and the formation of new combinations may result in acids, alkalies, alcohols, alkaloids, ptomains and many other combinations, some very poisonous and some innoxious, according to the fungus which produces it. Those which prey upon the human body will simply decompose it and convert it into its elements, always with the production of a bi-product which may be of a most virulent variety possible. It may be a toxine. For instance one-fifth of a grain of strychnine may be fatal, but if we take the bi-products of one of these organisms, the fatal dose would be represented thus, .00000001 of a grain. The poisoning in ice cream is usually caused by the growth of one of these organisms. Take the most common of all fungi, the yeast plant. What is the chemistry of its domestic use? A woman takes flour, which is starch and which may be converted into a form of sugar, glucose. She mixes it with water, adds the yeast plant and covers it up and sets it down before the stove to give it a proper degree of warmth. The yeast plant grows and gives off as bi-products, alcohol and carbon dioxide. Wherever one of these cells has grown we get this alcohol and carbon dioxide.

The $CO_2$ distends the dough and causes the rising of bread. When just right, the housewife puts it in the oven, fixes the dough, cooks it, and at the same time kills the yeast plant. If the bread falls, it is because the gas escapes before the dough is fixed by cooking.

Some of the micro-organisms grow in the mouth and decompose the food debris that lodges bewteen the teeth, with the evolution of an acid, and that is the central fact of the Miller theory. Into the mouth is taken constantly foods which lodge in the depressions or sulci of the teeth or somewhere between the teeth. This is infected with the fungi and decomposed with the formation of lactic acid which unites with the calcium salts of the tooth and thus a cavity is formed. The organisms penetrate the dental tubuli in advance, there forms more acid and this dissolves out the structure of the teeth until they crumble away as we so often see them. The organic portion of the tooth is left, perhaps of a kind of a leathery consistency until this in turn is decomposed by yet other fungi.

Miller obtained more than 100 different organisms from the human mouth, and amongst these was one impossible to be distinguished from the cholera bacillus. Cholera, that terrible disease, is but the growth of a single organism, so small that it can be seen with but the strongest microscopes. It developes in the body and produces its poison, the effect of which we call cholera.

In the slide here shown, the micro-organisms have penetrated the enamel and infected this area. Here is another that shows the beginning of infection in the dentine. They have entered the dentinal tubuli and decomposed the dental fibrilli with the formation of an acid which has dissolved the lime salts and enlarged the tubuli. In the next slide we see one a little more infected. Organisms have penetrated deeper into the tubuli and there is a much larger proportion of them affected with the commencement of the breaking down of the dentine. In the next slide this is seen in a greater degree. All the dental tubuli have broken down and have become merged together in a dissolution of inter-tubular substance. You can even see the effect of pene tration of the micro-organisms into the dental tubuli, how they have been broken down and the whole thing is diseased.

Here in another slide which is broken down still further with the formation of cavities, while here in this you see the bacilli. If you have followed me closely you can comprehend the process up to the point of the formation of the carious cavity, to the breaking down of the tubuli.

The next one is one of Miller's slides. You see the penetration of the organisms into the tubuli. It is quite powerfully magnified and we see the different micro-organisms. In this next one we have a breaking down and beginning formation of cavities, you see a cavity formed here, from the breaking down of several into one. This is another one very highly magnified which shows the breaking down. Here is another one cut diagonally, and here you see the formation of cavities of decay from another standpoint. Here is one melted down completely, you can see the steps and various processes of this breaking down and the formation of cavities with decomposition of inter-tubular substance.

Miller produced an artificial caries aside from vital connections, and subjected it to nearly the same circumstances as he found in the mouth and produced a caries which no man could determine from another. The slide now on the screen is the first from which Dr. Miller demonstrated the fact. When he demonstrated this, men were obliged to accept the fact that micro-organisms were the cause and not the results of diseased action. This slide of artificial caries is practically the same as the others. The melting away of the dental tubuli has commenced. Here is another one, the micro-organisms penetrating into the dental tubuli precisely as in natural caries.

There is one in which there is decay of pulp canal. You can see how it is infected; you observe the condition in which the root canals are sometimes found—all broken down and quiet decayed.

Magitot's theory in connection with decay of the teeth, was, that nature sent out a dense deposit to protect the tooth from further decay. You can see from this slide that the refractive power of this area is very different from any other and is supposed to be caused by this advanced deposition of calcareous deposits.

The advance of caries in the lower animals does not essentially differ from that of man. This slide shows the progress of decay in the tooth from a sea-cow or dugong, and is substantially the same as in man. Here is another practically the same thing, but shows breaking down into a canal.

I now show you a picture of the man to whom the world of science, and especially dental science, is so greatly indebted, Prof. W. D. Miller, seated in his laboratory in Berlin, and with this will close the series of views with many thanks to this large audience for its patience in listening to me at such great length.

